# Subgraph “Backbone” Analysis of Dynamic Brain Networks during Consciousness and Anesthesia

**DOI:** 10.1371/journal.pone.0070899

**Published:** 2013-08-15

**Authors:** Jeongkyu Shin, George A. Mashour, Seungwoo Ku, Seunghwan Kim, Uncheol Lee

**Affiliations:** 1 Department of Physics, Pohang University of Science and Technology, Pohang-si, Gyeongsangbuk-do, South Korea; 2 Department of Anesthesiology, University of Michigan Medical School, Ann Arbor, Michigan, United States of America; 3 Department of Anesthesiology, Neuroscience Graduate Program, University of Michigan Medical School, Ann Arbor, Michigan, United States of America; 4 Department of Anesthesiology, Asan Medical Center, University of Ulsan College of Medicine, Ulsan, South Korea; 5 Department of Physics, Pohang University of Science and Technology, Institute for Edge of Theoretical Sciences, Pohang-si, Gyeongsangbuk-do, South Korea; Laureate Institute for Brain Research and The University of Oklahoma, United States of America

## Abstract

General anesthesia significantly alters brain network connectivity. Graph-theoretical analysis has been used extensively to study static brain networks but may be limited in the study of rapidly changing brain connectivity during induction of or recovery from general anesthesia. Here we introduce a novel method to study the temporal evolution of network modules in the brain. We recorded multichannel electroencephalograms (EEG) from 18 surgical patients who underwent general anesthesia with either propofol (n = 9) or sevoflurane (n = 9). Time series data were used to reconstruct networks; each electroencephalographic channel was defined as a node and correlated activity between the channels was defined as a link. We analyzed the frequency of subgraphs in the network with a defined number of links; subgraphs with a high probability of occurrence were deemed network “backbones.” We analyzed the behavior of network backbones across consciousness, anesthetic induction, anesthetic maintenance, and two points of recovery. Constitutive, variable and state-specific backbones were identified across anesthetic state transitions. Brain networks derived from neurophysiologic data can be deconstructed into network backbones that change rapidly across states of consciousness. This technique enabled a granular description of network evolution over time. The concept of network backbones may facilitate graph-theoretical analysis of dynamically changing networks.

## Introduction

General anesthesia rapidly modulates levels of consciousness and has been suggested to be a useful tool for the study of consciousness [1], [2]. The precipitous state transitions across loss and recovery of consciousness provide a unique opportunity to study dynamic brain network behavior.

The reduced level of consciousness during anesthesia is associated with topological changes of functional connectivity in the brain [3]–[8]. The disruption of functional connectivity and suppression of metabolic activity are common features demonstrated across numerous neuroimaging studies. However, the limited temporal resolution of conventional brain imaging techniques restricts such studies to topological rather than temporal changes, which may be critical to mechanisms of general anesthesia. Recent studies of single unit recordings and local field potentials in humans have demonstrated the importance of dynamic temporal changes of neural networks during general anesthesia [9].

To examine the spatial and temporal properties of functional brain connectivity simultaneously, we introduce a novel concept, termed “dynamic network backbones.” The dynamic network backbone allows us to measure the statistical relevance of subgraphs in a network and to trace the dynamics of subgraphs by extending the concept of a network motif [10] to the temporal domain.

A subgraph of network N is a graph whose nodes and links are included in N. A network motif is a subgraph pattern that occurs with greater frequency than in a random network. Conventional structural and functional network motifs mainly relate to topological characteristics such as nodes and connections in the network. Motifs quantify anatomical building blocks and elementary functional processing modes of a brain network. These small network building blocks provide insight into the rules governing the global brain network [10], [11].

To expand the motif into the time domain, we generated a network time series from a windowed EEG dataset and then evaluated the statistical significance of the possible subgraphs for a given network time series. If the occurrence of a subgraph is statistically significant, we define the subgraph as a temporal backbone of the dynamic brain network. The dynamic network consists of numerous temporal backbones and their configuration reflects the spatial and temporal properties of a functional brain network. The study of temporal backbones may provide unique information about the neural correlate of general anesthesia in the time domain. Furthermore, this method will be useful to study the significant change of network configurations across various states of consciousness induced by general anesthesia.

In the current study, we used two distinct anesthetics to modulate the level of consciousness. Using the dynamic network backbone method we investigated the number of network backbones that appeared, diminished and returned across different states of consciousness. In particular, we investigated the characteristic temporal evolution pattern for each network backbone around the state transitions and the distinctive effect of two types of anesthetics on the temporal evolution of subgraphs.

We hypothesized that there would be typical network backbones corresponding to the conscious, unconscious and recovery states. According to information integration theory, the repertoire of possible brain states would be reduced after loss of consciousness and would return after recovery [12]–[14]. We demonstrate a reduced number of network backbones after anesthetic-induced unconsciousness, as well as a complex interplay of both “constitutive” and “variable” network backbones.

## Materials and Methods

### Subjects

The EEG data were originally gathered for a prior study of the frontoparietal system [15]. The Institutional Review Board (IRB) of Asan Medical Center approved this study in human volunteers. After IRB approval and written consents from the participants, eighteen patients scheduled for elective abdominal or breast surgery (n = 18, male/female = 8/10, American Society Anesthesiologists Physical Status I and II, age 29–66 years) were enrolled in the study. Two different types of anesthetics were administered to patients, and eight channel EEG was recorded. The patients' state of consciousness was separated into five states (baseline consciousness, induction of anesthesia, general anesthesia, recovery from general anesthesia, full recovery).

### Anesthetic administration

Patients received no sedative or other medications before induction of anesthesia. One of two anesthetic regimens was randomly selected and administered to patients: (i) Propofol: target controlled infusion of 2.0 mg/ml was started and increased at a rate 1.0 mg/ml per 20 s until loss of consciousness (LOC) for nine patients; or (ii) Sevoflurane: 2 vol% was started and increased at a rate 2 vol% per 20 s until LOC for the other nine patients. Time to LOC was determined by checking every 5 s for the loss of response to verbal command (“open your eyes”).

### EEG recording

EEG was recorded at eight monopolar channels in the frontoparietal region (Fp1, Fp2, F3, F4, T3, T4, P3 and P4 referenced by A2, which followed the international 10–20 system for electrode placement) by a WEEG-32 (LXE3232-RF, Laxtha Inc., Daejeon, Korea) to study frontal-parietal connectivity originally with a sampling frequency of 256 Hz.

Electromyogram (EMG) was concurrently recorded at four bipolar channels (bilateral frontalis and temporalis muscle) by a QEMG-4 (Laxtha Inc., Daejeon, Korea) with a sampling frequency of 1024 Hz. The EEG and EMG recordings were divided into five monitoring epochs: (i) baseline, 5 min before anesthetic induction; (ii) induction, from start of anesthetic induction to LOC; (iii) anesthetized state, 5 min after LOC; (iv) recovery, from the end of anesthesia to recovery of consciousness (ROC); (v) post-recovery, 5 min after recovery in the Post-Anesthesia Care Unit. Fourier-based bandpass filtering (zero-phase forward and reverse filtering with Blackman-Harris window function) (0.5–35 Hz for correlation calculation, Delta: 0.1∼4.0 Hz, Theta: 4.0∼8.0 Hz, Alpha: 8.0∼12.0 Hz, Beta: 12.0∼30.0 Hz for phase locking value calculation) was applied to EEG data before the network backbone analysis.

### Network time series and dynamic network backbones

**Figure 1:**
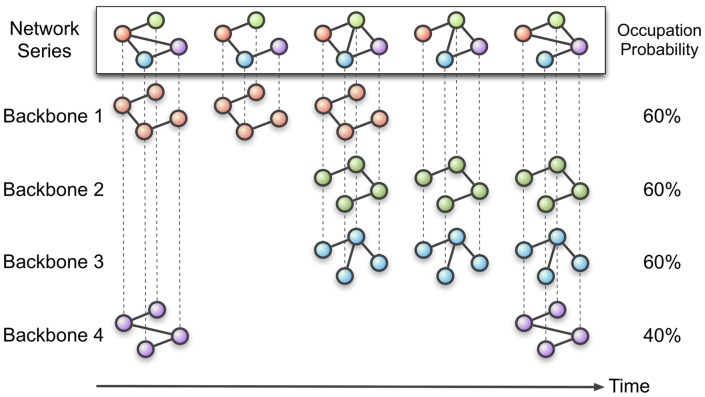
Extracting network backbones from a network time series. This diagram illustrates how to extract backbones and their dynamics from a network time series. The first row is a series of networks constructed from segmented EEG data. The other rows are network backbones extracted from the network time series. The network backbone persists for at least one EEG epoch. The number of links for a backbone is fixed as the smallest network size (3 for illustration) appearing in the given network time series. The occupation probability indicates the proportion of time windows in which specific network backbone appears over the given network time series.

**Figure 2:**
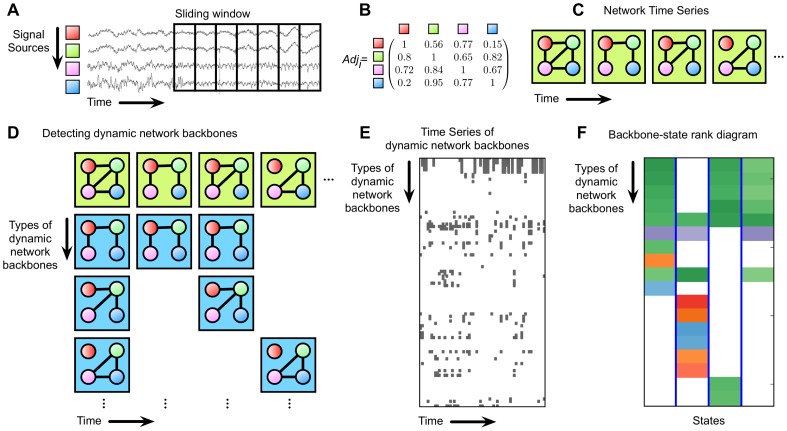
Scheme for extracting network backbones from an EEG time series. a) Segmenting EEG time series into small time windows (moving window method). b) Construct adjacency matrix by calculating mean phase coherence or Pearson correlation coefficient with zero lag among EEG channels at each window. c) Generate dynamic network time series. d) Identify dynamic network backbones. With dynamic network backbones, draw e) the time series of dynamic network backbones and f) the rank-state diagram.

The network time series is produced with EEG data fragmented using the moving window method and the statistically significant subgraphs are extracted from the network time series pool. Figure 1 and Figure 2 illustrate the procedure for extracting the network backbones. First, extract possible subgraphs from network time series. Second, count the number of their appearances through time. The subgraphs with statistically significant appearance compared with their randomized set (see *‘Statistical analysis’*) are defined as the network backbones, and the collection of them is the network backbone set.

The decomposition of a network into its modules in spatial terms is regarded as a *network motif* and *motif fingerprint.* Network backbones can be considered to be a temporal extension of the network motif approach. Backbones are derived from the series of networks, in which specific links are maintained across windows. It is distinguishable from the static network motif, which has a frequent spatial appearance compared with that of randomized networks. The network backbone is related primarily to temporal duration, while the motif fingerprint is based on spatial appearance. In this study, the network time series was generated from EEG by using a moving window method (window size: 6 second, moving size: 250 millisecond). At each window, the adjacency matrix was constructed by calculating coherence measure. Pearson correlation coefficient (0.5∼35 Hz range) and mean phase synchrony[16] (delta, theta, alpha, beta bands) were used independently in order to check the consistency. Mean phase synchrony (phase locking value) is defined at time *t* as the average value
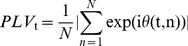
(1)where *N* is the number of sample datasets and 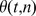 is the phase difference 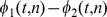. Phases are calculated by Hilbert transformation.

If a pair of channels whose phase synchrony is significantly higher than that of the surrogate datasets, the pair of channels was deemed to be functionally connected (See ‘Statistical analysis’). The parameter set including window size and threshold for producing binary network time series was chosen to maximize the diversity of networks at each state. Therefore, the entire process for constructing the network time series is non-parametric.

The network at time *t* is 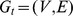, where V and E are nodes and edges in the network 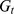, respectively. The subgraphs are 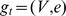, 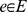. For a fully connected network, the subgraph can be labeled as
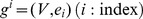
(2)


At time *t*, *module appearance* 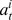 is defined as a binary number such that 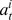 equals 1 if 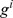 appears at time *t* and otherwise 0. The probability *p_i_* of each module from a series of networks is defined as follows. If the number of data points is *T*, *occupation probability* 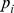, which is the probability of a specific network module appearing over the whole series of networks is
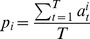
(3)


If a network at time *t* has *n* nodes and *h* distinct links, the number of possible modules is
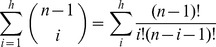
(4)


Since the total space of the modules is too large to compute efficiently, we fixed the module size at 4 (4 edges) in this study. This module size was determined by the smallest number of links in the series of networks for our EEG data, in order to guarantee that each window had at least one backbone candidate.

### Parameter estimates for functional connectivity

At each window, we derived a correlation matrix 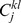 (*k, l*: channel index, *j*: window index) by calculating the correlation coefficient or phase synchronization index among EEG channels. A correlation matrix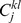 was converted to a binary form adjacency matrix
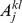
with threshold *h* (; 0 if 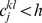 and otherwise 1). The size of network ensemble (*n*) was defined as the number of unique adjacency matrices (excluding redundant adjacency matrices) in the whole network time series. All parameters for these connections (threshold (*h*), window size(*d*) and moving size (*g*)) were determined with the parameter set that provides the largest network ensemble size (*n*), in other words, providing the most information on connectivity for a given multivariate data set. For our EEG data, the window size of 6 seconds and window moving size of 250 milliseconds consistently provided the largest network ensemble. However, the threshold for connection (*h*) was variable for states.

### Extracting the dynamic network backbones from a network series

**Figure 3:**
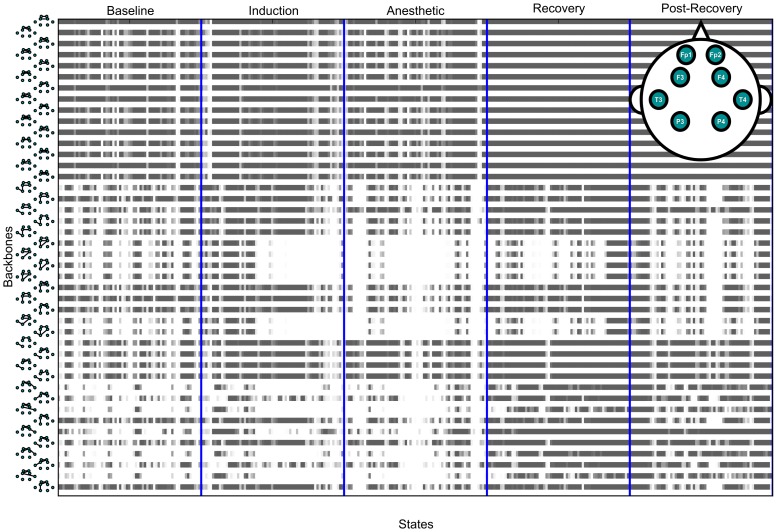
An example of dynamic network backbones from propofol-induced patients. The network backbones are sorted in the ascending order of the occupation probability in the baseline state and their occupation pattern throughout states. New network backbones emerge after the state transitions across five anesthesia stages. Each stage is divided by a blue vertical line. The black dots indicate the appearance of a network backbone.

After generating the largest network ensemble for a given EEG data set, we extracted the network backbones. First, we identified the smallest number of links (*m*) in the whole series of networks and then extracted all possible subgraphs with *m* links 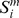 (*i*: link tuple index) in the series of networks. The number of possible subgraphs with *N* nodes and *m* links is *(N-1)!/m!(N-m-1)!*. To determine network backbones, we calculated the occupation probability 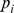 of 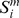 and took *L* highly ranked 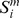 based on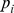. For instance, if a network backbone has an occupation probability of 1, it indicates that the backbone can be found throughout the entire network series. In this study, we focused on the temporal evolution of *L* highly ranked network backbones, assuming that highly ranked network backbones represent the temporal behavior of the whole network ensemble. The configuration of network backbones for each window was represented with binary symbols (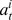, *i*: backbone index, *t*: window index, and 1: present, 0: absent in a window). Thus, the total network time series *G* is able to be converted into a series of binary symbols of all network backbones (visualizing “1” with black dot in Figure 3). Finally, we can use the binary form series of network backbones in order to study the temporal evolution behavior of each network backbone.

### Algorithm for reducing computation time

The computation time for detecting all motifs from a network depends on the size of network. There are many algorithms for detecting motifs with improved computation speed. However, the previous algorithms do not work for detecting dynamic network backbones because the motif does not incorporate the node indices, which are crucial for this process.

**Figure 4:**
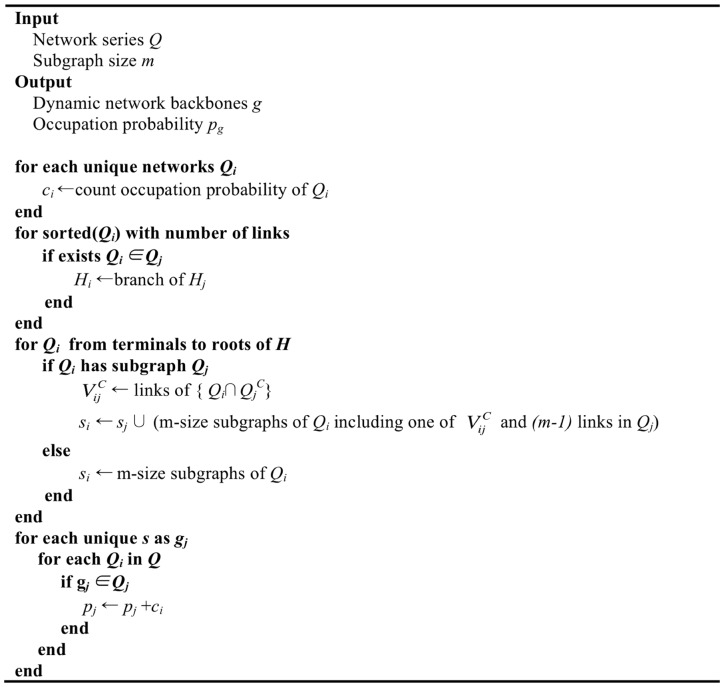
Pseudo-code of dynamic network backbones detection algorithm.

To reduce the computation, we used the hierarchical structure of subgraphs of network ensembles. If a network A is a *super*graph of network B, the *sub*graphs of the network B also become the *sub*graphs of network A. Thus, the basic ideas for reducing computation are: (1) construct the hierarchy of networks by their *super*graph-*sub*graph relationship, and (2) do not count the *sub*graphs that were already counted in the smaller networks in order to avoid duplicate extraction (See Figure 4 for pseudo-code). By removing the duplicate detection loops for the *sub*graphs of larger networks, we can reduce the computation time by O(ln N). In practical cases, the measured brain state (e.g. baseline, anesthetized) is a stationary state, which guarantees that the number of unique networks converges regardless of network time series length. As a result, the time complexity is reduced by O(N). Overall, the time complexity of the whole procedure is reduced by O(N ln N).

### Similarity of network backbone configurations across states and anesthetic groups

To quantify inter- and intra-subject similarity of network backbone configurations across the five states of consciousness, we measured the cosine similarity between network backbone configurations. Cosine similarity is defined as
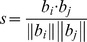
(5),where b_i_ and b_j_ are the network backbone sets for state *i* and *j*. For example, if there are three types of backbones in a network time series, type 1 and 3 appear in the state *i*, and type 2 and 3 appear in the state *j*, then *b_i_* and *b_j_* are denoted as {1, 0,1} and *b_j_* = {0,1,1}, respectively. The cosine similarity *s* is



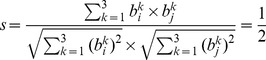
(6)The range of cosine similarity is from 0 to 1; 1 indicates the same configuration, otherwise, 0 indicates a completely different configuration.

To quantify the similarity of backbone configurations across states, we extracted the 60 most probable network backbones from each state. For direct comparison, the indices of network backbone types are matched over all backbone configurations, filled with 0 for nonexistent backbone types. It is notable in this study that a similar network backbone configuration indicates similar *temporal* configuration of network backbones, which is distinct from similar *spatial* configuration.

### Statistical analysis

The functional connections between EEG channels were determined by a comparison with a randomized data set. Randomized data sets were created by random phase shuffling while retaining the same power spectrum using the AAFT method [17]. 40 randomized data sets were created for each time window. If the phase synchrony or the correlation coefficient between two EEG channels was significantly deviated from that of randomized data (p<0.05 with one paired t-test), we deemed the EEG channels to be functionally connected. This comparison was performed to avoid the problem of spurious correlation/phase synchrony measurements that can result from lower frequency dominant power spectrum in the anesthetized state [18]. Through this process, the series of weighted networks *G_i_* were generated from EEG data.

**Figure 5:**
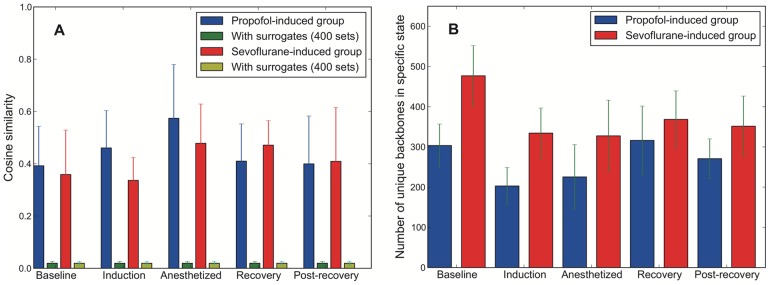
The inter-subject similarity of network backbone configurations for different states and anesthetic groups. a) The similarities of network backbones profiles among subjects were measured by cosine similarity. The network backbone profile for a subject was constructed with 400 backbones and was compared with others in five states of each anesthetic group. To test the significance of the similarity of network backbones, the 400 surrogate data sets for each subject were generated and the similarity of network backbones was calculated (Green bars). Errorbar indicates standard deviation of the similarities for all pairs of subjects. b) The number of unique network backbones for each state (from top 1600 backbones). Errorbar indicates standard error of subject variability. Unique backbones are the backbones that appear only in a certain state for each anesthetic group. The higher number of unique backbones in the baseline state of the sevoflurane group indicates that the other states share fewer backbones with baseline state compared to the propofol group.

The inter-subject variability of network backbones and the significance of dynamic network backbones were tested. We chose the backbones commonly found over all subjects (>77% of subjects) as dynamic network backbones (common network backbones). The significance of dynamic network backbones was assessed by comparison with the surrogate data. For each data set, we generated 400 surrogate time series and extracted the network backbones. Kruskal-Wallis H-test was applied to the original and the surrogate data sets. The significance level of the test was set as p<0.001. For every test set, p-values were less than10^−6^, which strongly rejects the null hypothesis (See Figure 5). All statistical tests are performed with scipy statistics module (0.10) and Kruskal-Wallis H-tests were checked with a statistical toolbox in MATLAB® R2011a.

### Materials

MATLAB® version of our dynamic network backbone extraction codes is available at http://github.com/inureyes/network-backbone-toolkit.

## Results

### Configuration of dynamic network backbones for each state and anesthetic group

We investigated the configuration of network backbones across various states of consciousness and between two anesthetic groups. Figure 3 demonstrates an example of the time course of dynamic network backbones during the experimental period. The complex configurations of network backbones and the transient temporal reorganization are presented across states. Figure 5a presents the mean cosine similarity of network backbones among subjects for the two anesthetic groups. 400 network backbones were extracted from each subject and the inter-subject similarities within each group were measured. About 40% of network backbones (out of 400) are shared among subjects, and the number of common network backbones is decreased during anesthesia (Kruskal-Wallis H-test, statistics: p<0.001). Figure 5b demonstrates the number of state-specific network backbones. The state-specific (unique) backbones are determined by comparison with the backbones in the other four states. If a certain backbone in a specific state appears in the other four states, the backbone is excluded from the unique backbones. It is notable that each state has specific backbones that are unique and that general anesthesia is associated with a reduction of state-specific backbones (Mann-Whitney U test, statistics: p<0.01). It must be emphasized that the determination of a state-specific backbone is dependent on all states analyzed. Thus, the observed differences in the specific backbones of the “Baseline” state between the two anesthetic groups does not mean that the two groups were different at baseline, because this state was not analyzed in isolation. Rather, backbone differences in the anesthetized or recovery state will influence the determination of what is unique in each group in the baseline state.

We summarized the configuration of dynamic networks for each state and two anesthetic groups. Table 1 presents the number of unique networks and common network backbones that appeared in the pooled data for both anesthetic groups (i.e., propofol and sevoflurane groups). 1196 network configurations are possible from 300 sec state size and 0.25 second moving window size, and approximately 21% of all possible configurations appear (Baseline: 276.33±76.09, Induction: 249.22±63.75, Anesthetized: 231.67±80.67, Recovery: 262.33±87.34, Post-recovery: 242.33±75.10 for propofol-induced patients) in the data. About 79% of possible network configurations did not occur in the reconstructed brain networks, suggesting that the temporal organization of dynamic networks is not a random process, but rather strongly constrained (Kruskal-Wallis H-test with 300 surrogate set, statistics: p<0.001).

**Table 1:** Brain networks and dynamic network backbones for nine subjects and each anesthetic (cross-correlation network).

**States**	5	**Subject#**	9	**Backbone Size**	4
**State size**	300.0 sec.	**Window Size**	6.0 sec.	**Moving Size**	0.250 sec.

PROPOFOL					
	Baseline	Induction	Anesthetized	Recovery	Post-recovery
**Threshold**	0.5944±0.0984	0.5144±0.0826	0.4255±0.1661	0.4322±0.1699	0.5822±0.0940
**Network ensemble size**	276.33±76.09	249.22±63.75	231.67±80.67	262.33±87.34	242.33±75.10
**Number of common backbones**	428	526	971	494	288
**Max. occupation probability**	0.9710±0.0457	0.9899±0.0130	0.9964±0.0068	0.9807±0.0270	0.9485±0.0878
**Min. occupation probability (top-12)**	0.9314±0.0998	0.9575±0.0332	0.9921±0.0152	0.9597±0.0842	0.9214±0.1252
**(top-60)**	0.8698±0.1607	0.8817±0.0542	0.9625±0.0840	0.9267±0.0701	0.8568±0.1639
**(top-400)**	0.7171±0.2419	0.6746±0.1161	0.8487±0.1643	0.7695±0.1522	0.6839±0.2710

SEVOFLURANE					
	Baseline	Induction	Anesthetized	Recovery	Post-recovery
**Threshold**	0.5767±0.1246	0.5789±0.1295	0.5222±0.0895	0.4611±0.0929	0.5378±0.0917
**Network ensemble size**	307.00±89.68	276.11±78.94	261.44±74.18	286.00±64.87	269.11±75.70
**Number of common backbones**	279	323	563	443	416
**Max. occupation probability**	0.9667±0.0485	0.9124±0.1228	0.9965±0.0100	0.9857±0.0269	0.9726±0.0538
**Min. occupation probability (top-12)**	0.8887±0.1356	0.8587±0.1558	0.9882±0.0316	0.9672±0.0359	0.9441±0.0866
**(top-60)**	0.7947±0.1815	0.7653±0.1934	0.9720±0.0510	0.9297±0.0710	0.9118±0.1294
**(top-400)**	0.6370±0.2067	0.5826±0.1930	0.7766±0.1745	0.7702±0.1218	0.7738±0.1807

A unique network is a network with a distinctive connection structure in the given network time series. Network ensemble size is the number of unique network structures appearing in the data. The number of common backbones is the number of commonly found backbones across most subjects (>77%, 7/9 subjects in this study). *maxP* denotes the maximum occupation probability that the network backbones can have for the pooled data. *minP* denotes the minimum occupation probability that the network backbones can have. For example, *minP*(12) means the minimum occupation probability of the top 12 backbones. The *minP(#)* demonstrates how slowly the occupation probability was reduced as the number of network backbones considered increases. Note that in the anesthetized state the number of unique networks was reduced from baseline, indicating more frequent repetition of the same network and network backbone.

The minimum occupation probabilities of top-*n* network backbones (n = 60, 400) are significantly increased after anesthesia (Mann-Whitney U test statistics, p<0.01, if significant). The decreased number of unique networks and increased number of common network backbones implies a more monotonous pattern. The higher occupation probability in the anesthetized state reflects the lack of variety in the spatial and temporal configurations, which is what would be predicted.

### Categorization of dynamic network backbones based on rank diagram

**Figure 6:**
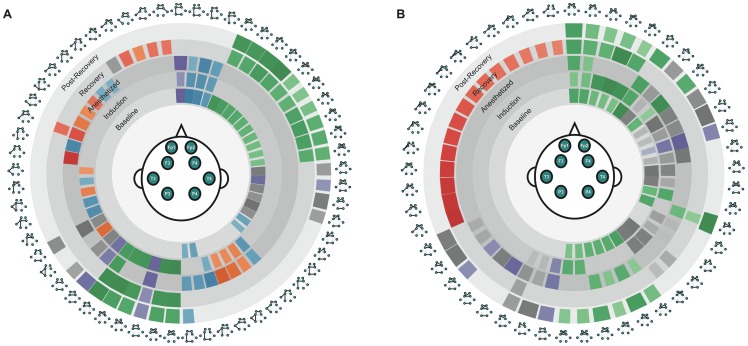
Demonstration of backbone-state rank diagram for each anesthetic group. a) Propofol patient group and b) Sevoflurane patient group. The network time series are constructed by Pearson correlation coefficient with zero lag (0.5∼35 Hz) between EEG channels. Only the top 24 dynamic network backbones are shown in these figures (for the sake of readability). The network backbones were sorted from constitutive to variable and state-specific backbones in descending order, clockwise from top center. The wider patch represents a higher occupation probability of the backbone in a network time series. The network backbones were categorized into groups based on the connection, and presented with colors: prefrontal-frontal connections (green), (pre)frontal-parietal connections (blue), intra-parietal connections (gray), inter-hemispheric connections (purple) and temporal-parietal connections (orange).

**Figure 7:**
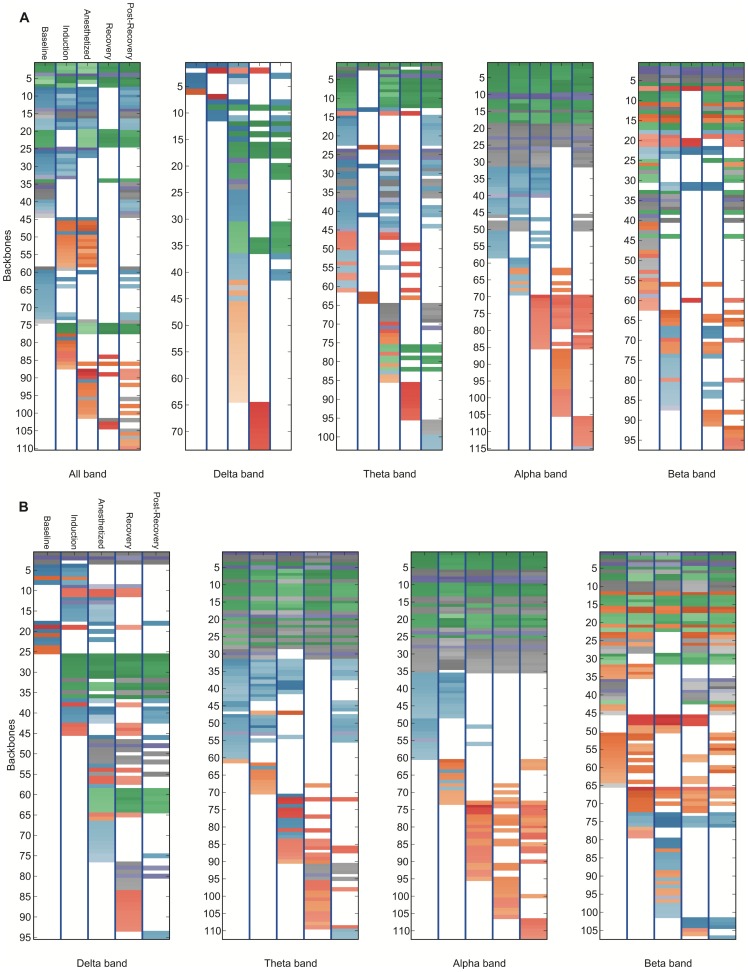
Bandwidth-specific backbone-state rank diagram for the propofol group. The network time series are constructed (a) by Pearson correlation coefficient with zero lag (all bands: 0.5∼35 Hz, delta: 0.1∼4.0 Hz, theta: 4.0∼8.0 Hz, alpha: 8.0∼12.0 Hz, beta:12.0∼30.0 Hz) and (b) by mean phase coherence (band-specific) between EEG channels. 4-link backbones are extracted. The top 60 dynamic network backbones are shown in these figures. The network backbones were sorted from constitutive to variable and state-specific backbones in descending order. The darker color means a higher occupation probability of the backbone in a network time series. The network backbones were categorized into groups based on the connection, and presented with colors: prefrontal-frontal connections (green), (pre)frontal-parietal connections (blue), intra-parietal connections (gray), inter-hemispheric connections (purple) and temporal-parietal connections (orange).

**Figure 8:**
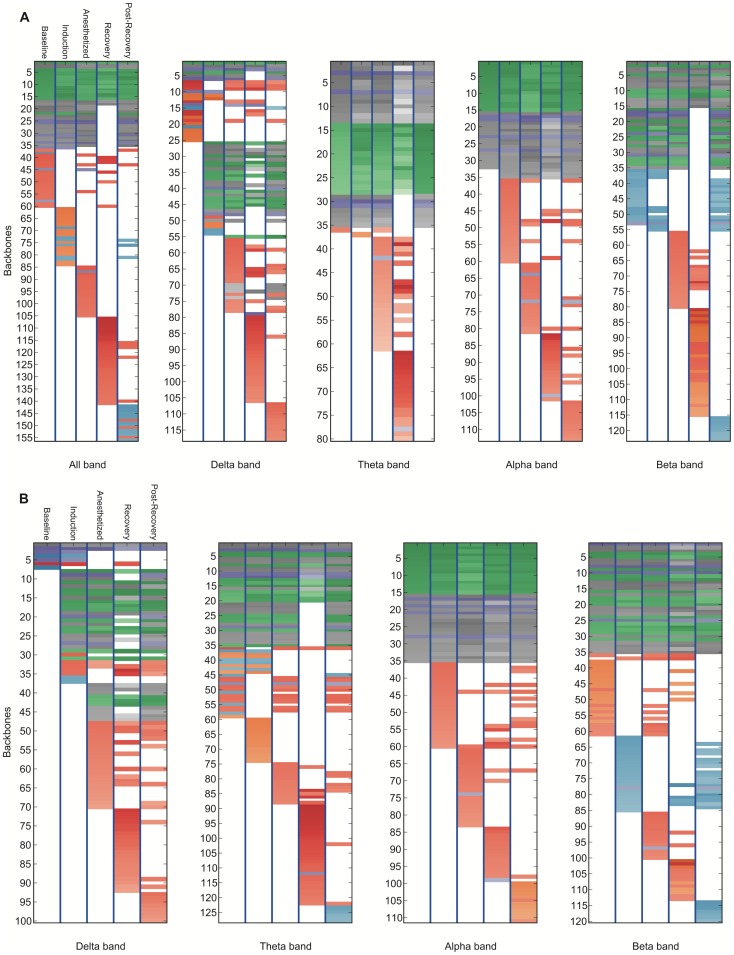
Bandwidth-specific backbone-state rank diagram for the sevoflurane group. The network time series are constructed (a) by Pearson correlation coefficient with zero lag lag (all bands: 0.5∼35 Hz, delta: 0.1∼4.0 Hz, theta: 4.0∼8.0 Hz, alpha: 8.0∼12.0 Hz, beta:12.0∼30.0 Hz) and (b) by mean phase coherence (band-specific) between EEG channels. 4-link backbones are extracted. The top 60 dynamic network backbones are shown in these figures. The network backbones were sorted from constitutive to variable and state-specific backbones in descending order. The darker color means a higher occupation probability of the backbone in a network time series. The network backbones were categorized into groups based on the connection, and presented with colors: prefrontal-frontal connections (green), (pre)frontal-parietal connections (blue), inter-hemispheric connections (purple), intra-parietal connections (grey) and frontotemporal connections (red). Note that frontotemporal circuits are activated during the recovery process.

We categorized the network backbones based on the occupation probability and the connection structure. By categorizing many backbones, we can easily study the relationship between the states and the network backbone configuration. The dynamic network backbones were ranked by their occupation probability at each state, which we term a ‘backbone-state rank diagram.’ The ranked backbones were categorized by the connection structure, which is denoted by different colors. The color intensity indicates its rank. Figures 6–8 present the rank diagrams of network backbones for two anesthetic groups, which were constructed using the common backbones of the pooled data of nine subjects for each group.

Based on the rank diagram, we identified “constitutive” network backbones that are present during all states, and “variable” network backbones that emerge or disappear in specific states. The rank diagram for propofol-induced anesthesia (Figure 6a and Figure 7) demonstrates that some network backbones linking the prefrontal and frontal regions (green) persisted across state transitions. By contrast, network backbones that linked the left frontal and left parietal regions (blue) appeared only transiently (induction-anesthetized, post-recovery). Network backbones that linked the left and right hemispheres (purple) were reduced in the recovery stage. Temporal-parietal backbones (orange) appear in states affected by anesthetics (induction, anesthetized and recovery state). Some types of network backbones that linked the prefrontal and frontal regions appeared only at baseline and recovery state, and then continued until the end of recording.

In Figure 6b and Figure 8, the rank diagram of sevoflurane anesthesia demonstrated more complex network dynamics compared to that of propofol. However, the network backbones linking the prefrontal and the frontal regions were persistent across all states as was found with propofol. The number of unique network backbones corresponds to the diversity of networks. In our study, sevoflurane-induced patients typically showed more diverse backbones than propofol-induced patients. For instance, the sevoflurane rank diagram with the top 60 network backbones (all bands, cross-correlation network case) included 156 different types of common network backbones over the five states, compared to 110 types in the case of propofol (first rank diagram of Figure 7a and Figure 8a). This may reflect differences of anesthetic mechanism or possibly different levels of consciousness, as the two anesthetics were not given in equisedative doses.

### The similarity of network backbone profiles across states

Some network backbones are maintained while others constantly changed, reflecting a complex change of network backbone configuration along with the change of states during general anesthesia. To measure the similarity of network backbone configurations across states, the cosine similarity index was used with the top network backbones, as defined by occupation probability for each state.

**Figure 9:**
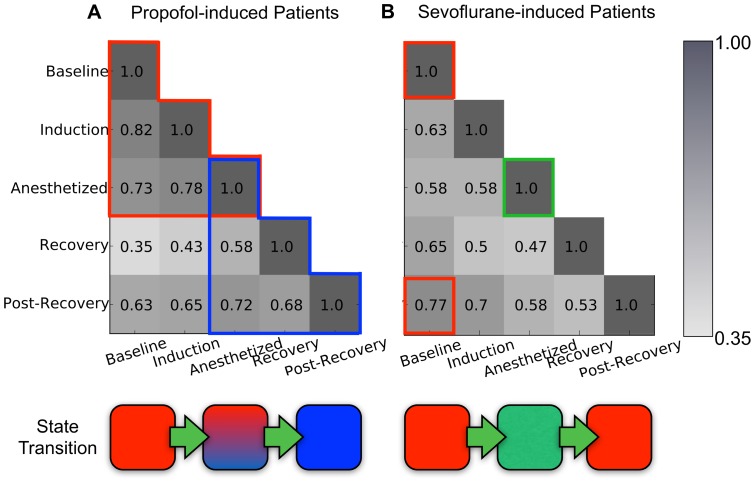
The similarities of network backbone configurations among the five states in the two anesthetic groups. (a) propofol and (b) sevoflurane. The similarity of the five states was measured with the 60 network backbones that have the highest occupation probability for each state. Darker color indicates higher similarity. The two transition states (induction and recovery) are dissimilar from one another in both anesthetic groups. The red and blue boxes in Figure 9a denote the higher similarities among states in the propofol group, which are not found in the sevoflurane group. The illustrations below the matrices present the distinctive recovery pathways for two anesthetic groups: for propofol, the network backbone configuration was not recovered, whereas it was for sevoflurane.

Figure 9 demonstrates the similarities of the top network backbones across five states: waking, induction, anesthesia, recovery and post-recovery. The top 60 network backbones were selected from the common network backbones of the nine subjects in each anesthetic group. The similarity *s* between the top 60 network backbones of two groups was measured as a normalized value (from 0 to 1) using cosine similarity. For two given network profiles, *s* = 1 denotes that two network backbone profiles are perfectly preserved and *s* = 0 means that the profiles are completely changed.

In Figure 9a, propofol induces a relatively slow change of network backbone profile after induction (*s* = 0.82) and general anesthesia (*s* = 0.73). After a large change at the recovery state (*s* = 0.35), only 63% of the network backbone profiles are returned to the post-recovery state. By contrast, sevoflurane produces relatively large changes at induction (*s* = 0.63) and anesthesia (*s* = 0.58), and smoothly returns to the original level of network backbone profiles up to the post-recovery state (*s* = 0.77) (in Figure 9b). In terms of the changing pattern, the loss of consciousness (baseline, induction and anesthetized states, within the red box of Figure 9a) and the recovery of consciousness (recovery and post-recovery, within the blue box of Figure 9a) are distinguishable in propofol-induced anesthesia.

The lowest cosine similarity is found in the comparison between the induction and the recovery state (*s* = 0.43 for propofol, *s* = 0.50 for sevoflurane), both of which are transition states from consciousness to unconsciousness, and vice versa. The distinctive configurations of network backbones for both transition states may reflect different state transition mechanisms for the loss and recovery of consciousness. Regarding the network backbone profile of the anesthetized state, the similarity with the other states was low (Figure 9a and Figure 9b).

### Robustness of network backbones from noise contamination

We tested the sensitivity of the algorithm to noise, since empirical datasets and clinical environments generally contain noise from various sources.

To test the robustness of network backbones against noise contamination, we simulated a network time series, which consists of 4,000 networks with 6 nodes and 38,000 random links (the average number of links is 9.5, with a range of 4∼15 links for each network). In this simulation, two noise parameters were introduced into the simulated networks: the strength of random rewiring and the proportion of noise contamination. The random rewiring was applied to 1% to 20% of links at each network. The rewiring range for the whole network time series was changed from 0.025% (1 network), 6.25% (250 networks), 12.5% (500 networks), 18% (720 networks), 50% (2,000 networks), and 75% (3,000 networks). After that, we measured how many of the original network backbones were extracted from the randomized network time series at each noise level. We repeated the simulation test 450 times and summarized the results.

**Figure 10:**
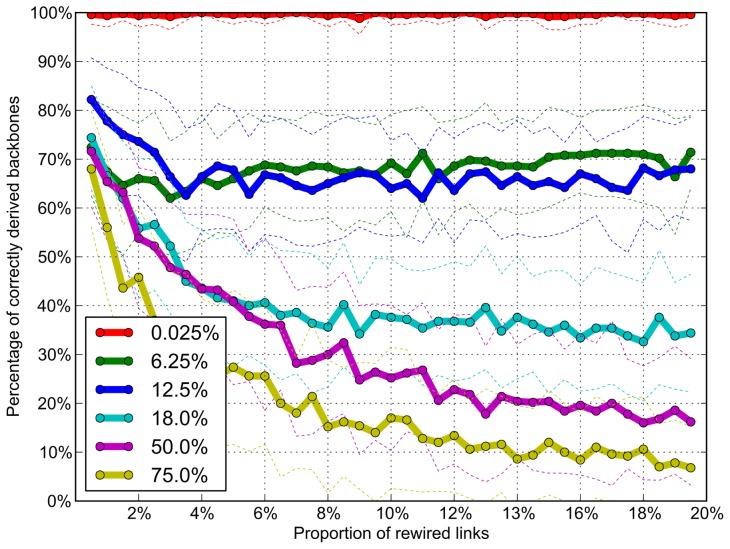
The robustness of network backbones in response to noise contamination. The noise effect was introduced into 450 simulated network time series in two ways: increasing the random rewiring ratio (from 1% to 20%) for each network and increasing the period of random rewiring (from 0.025% to 75%) for the sequential networks. Dotted lines indicate the standard deviation of tests. Each series has 4,000 sequential networks. Each network has 6 nodes and about 9.5 links on average. About 70% of the network backbones were preserved with the random rewiring ratio of up to 20%. However, the period of random rewiring more significantly affected the original configuration of network backbones. Each color corresponds to the period of noise contamination. The error bar indicates the standard deviation over the 450 simulated network time series.

Figure 10 demonstrates the robustness of dynamic network backbone method. About 70% of the original network backbones are preserved irrespective of the level of random rewiring up to 20%. The short-term noise contamination (6.25% and 12.5% in Figure 10) does not interfere significantly with the original backbones, even with strong random rewiring. On the contrary, relatively long term noise contamination (18%, 50% and 75%) significantly disrupts the original network backbones even with smaller random rewiring at each network. The robustness test shows that the noise contamination proportion in network time series is more influential on the original network backbone than the strength of random rewiring. This result is due to the method of extracting network backbones based on the occupation probability. The occupation probability is more strongly affected by the proportion rather than the strength of random rewiring for a short-term epoch. These findings suggest that applying proper noise filters to the time series is important to obtain precise network backbones, whereas sharp noises are negligible.

## Discussion

This study reports a novel technique for analyzing dynamic network changes across rapidly changing states of consciousness. The analysis of backbones revealed characteristic time evolution features of basic network elements and complex assembly patterns of network subsets during general anesthesia.

Our approach has several advantages: (1) The concept of dynamic network backbones expands the study of structural motif to motifs in the time domain. By definition, the significant temporal subgraphs (backbones) reflect the dynamic configurations of the network, which has not been reflected in the spatial motif. This is essential to the study of brain networks with state transitions accompanied by a change of network structure. (2) The dynamic network backbone method reveals new dynamic properties of brain networks (variable, constitutive and state-specific) for various states of consciousness. The dynamic properties of the brain network consistently appeared for two different connection measures (Pearson correlation coefficient and mean phase coherence), four frequency bands, and two anesthetic groups. This method can be applied to other fast state-transition data in order to study the temporal configuration of substructures in the brain network. (3) The method also revealed that even a small number of EEG channels produces abundant and complex network backbones. This is possible because the dynamic network backbone takes into account the time domain. Network study with a small number of nodes (e.g., channels) is an important advantage of this method. (4) The dynamic network backbone is robust after exposure to strong short-term noise contamination, which is common with empirical data from EEG. Because of these advantages, the dynamic network backbone approach is appropriate for studying functional circuits of both stationary state networks and rapidly changing networks with a small number of nodes.

With respect to general anesthesia, there are four main findings to this study: (1) Anesthetics significantly reduce the diversity and population of network backbones. (2) Different anesthetics produce different changing patterns of network backbones, possibly reflecting diverse molecular mechanisms. (3) Some network backbones are not affected by anesthetics (e.g., local connections such as prefrontal-frontal), while other backbones were variable across states (e.g., long-range connections such as frontal-parietal) [19], [20]. (4) The deformation of network backbone profiles at the loss and recovery of consciousness were unique to each anesthetic.

Theories of meta-stability and dynamical system models describe the dynamic coordination between different parts of the brain [21]–[23]. Meta-stability is a theory of how global integration and local segregation coexist in the brain. Several approaches have been attempted to measure meta-stability based on empirical data in different conscious states. These studies have suggested that different conscious states can be characterized by different hierarchical structures of simple patterns of brain activities, local or global functional connectivity, and their temporal evolution properties [22], [24]–[26]. The current study extends this line of investigation by introducing a method to study dynamic network changes across time. By using the network backbone method we were able to study quantitatively the change of hierarchical structure of brain networks across different states of consciousness induced by anesthetics as well as the distinct effects of two anesthetics.

General anesthesia simplifies the complex temporal evolution pattern of brain networks and reduces the population of network backbones, transforming diverse brain connectivity into a more restricted and consistent pattern. For both anesthetics, the number of network backbones was reduced during induction, and also reduced under anesthesia (See Table 1). The reduced complexity of EEG activities in the anesthetized brain has been demonstrated using various measures of entropy [27], [28], but this is the first reported quantification of the number of local functional network elements. It is important to note that our interpretations rest on an unproven assumption, namely that the prolonged duration of a network backbone implies a significant role in brain function. It is also notable that during anesthetic-induced unconsciousness, a limited reduction of network backbones took place, while the majority of network backbones in the brain were preserved. Furthermore, a number of backbones appeared during general anesthesia, some of which were state-specific and others that persisted throughout recovery. These data suggest that general anesthetics do not simply “turn off” certain networks or “turn on” others. Rather, there is likely a complex mosaic comprised of constitutive backbones that persist across all states, other backbones that appear to be specific for the state of consciousness and general anesthesia, and still others that appear during anesthesia and are maintained throughout the return of consciousness. The novel method of dynamic network backbone analysis may better reflect the diverse and dynamic changes of network structure during general anesthesia compared to current techniques of analyzing brain connectivity.

The constitutive and variable backbone types may be linked to specific neuroanatomical substrates, but given the low spatial resolution of the network this will require further testing with higher-resolution methods. Local connectivity structures, such as the prefrontal-frontal connections, tended to be constitutive, whereas relatively distant connectivity structures, such as the frontal-parietal, inter-hemispheric and frontal-temporal connections, represented variable backbones. The persistent frontal-prefrontal network backbones under anesthesia are consistent with our past study, in which the frontal and prefrontal network preserved its optimal network structure after anesthesia, whereas the parietal network was significantly disrupted [7]. The preserved network backbones indicated relatively less anesthetic effect on the frontal-prefrontal network after loss of consciousness. On the other hand, the reduction of network backbones involving the frontal-parietal connections (F3-P3) supports the hypothesis that the disruption of frontal-parietal networks is a neural correlate of anesthesia [1], [5], [7], [8], [15], [19], [29], [30].

Our findings are consistent with both magnetic resonance imaging and electroencephalographic studies from other research groups suggesting that local network connectivity can be preserved during general anesthesia, whereas longer-range connections are disrupted. The disruption of long distance functional connections has been suggested to be one neural correlate of anesthesia [19], a hypothesis that has been recently supported in humans [9]. Persistent brain networks during general anesthesia were reported in past fMRI studies [31], in which the network reflected the averaged hemodynamic response at a very low frequency range [32], [33]. A study using electrocorticography reported that variable cortical activities were superimposed on maintained functional architectures during propofol anesthesia [34]. In our study, network backbones with long range connections disappeared in the anesthetized state while the backbones with local connections were persistent, which is highly consistent with prior studies (Figures 6–8) [19], [31]–[33], [35], [36].

Finally, network backbone profiles were compared across states produced by both anesthetics. The overall patterns of similarity across states can be distinguished between the two anesthetics. Sevoflurane resulted in a larger deformation in the network backbone profiles of the induction and anesthetized states, compared to those of propofol. Sevoflurane also produced faster recoveries of the baseline profiles after return of consciousness and at the post-recovery state. However, the anesthetics were not necessarily maintained at equisedative concentrations with a common behavioral measure and thus it is unclear if backbone differences are related to the molecular mechanism of the drug or to different levels of consciousness. It should also be noted that the differences of network backbone profiles at the recovery and post-recovery stages between the two anesthetics could be affected by the differences of surgical procedures. Future studies designed specifically to compare the two drugs will have to be performed to characterize more precisely the differential effects on network backbone structures.

### Limitations

The present study has numerous limitations. First, this work considered only low pass filtered EEG data (<35 Hz) to avoid potential contamination of muscle artifact. Thus, the correlation results excluded higher frequency elements of EEG (in particular, gamma frequency band, 35–100 Hz), which may be important for consciousness. Second, eight EEG channels resulted in a reconstructed network with low spatial resolution. However, the goal of this study was to demonstrate proof-of-principle that the network backbone technique could be useful for assessing dynamic state changes. Further study with high-density EEG is warranted. Third, monopolar EEG recording of common reference (A2) could give rise to a volume conduction effect. We did not treat this potential problem in the network construction. Instead, we used strict criteria to determine connections by surrogate data. Fourth, extracting phases from EEG with a broad frequency range frequently provides incorrect phase information. To avoid this, only phase synchrony significantly deviated from a surrogate dataset, which has the same spectral contents, was deemed a functional connection. Fifth, heavy computation is required for extracting dynamic network backbones, compared to conventional network motifs [10], [11]. This was part of the motivation to use a network with limited nodes for the initial description and analysis of network backbones. The run-time complexity is *O(N^n^)*, where n is the size of subgraph. With multi-threaded code on a 12-core Intel Xeon x5520 processor, the computation time of extracting whole backbones for a single patient was about 15 minutes with 4-link backbones from 8 nodes and 6,000 epoch network time series; computation time increased to 120 minutes with the same condition but 21 nodes (from prior experiments not shown). Thus, at this time we cannot suggest that backbone analysis would be useful in a real-time clinical setting. If we assume that the time-series is measured from a stationary state, the alternative algorithm described in the Methods section can reduce the calculation time by *O*(*N*ln*N*) (see Methods). Sixth, the significant spectral power shift after anesthesia could produce a relatively higher signal to noise ratio (SNR). Thus, there is a potential for a higher SNR in anesthesia giving rise to a larger number of common backbones with higher occupation probability. However, it is difficult to evaluate the SNR level of EEG data and its effect on network backbones. To avoid this potential problem, we (1) identified functional connections only if they were significantly deviated from those of random data with the same power spectrum, (2) used phase coherence, which is less affected by linear mixing noise contamination, (3) tested the random effects produced by specific power spectra of the five states (Figure 5a) and (4) tested two different types of noise contaminations (Figure 10). Finally, it is important to note that the backbones described in this study are mathematical constructs that do not necessarily reflect neuroanatomical substrates or functional modules. However, our findings regarding prefrontal-frontal backbones and frontal-parietal backbones are consistent with other studies and other neuroimaging modalities.

## Conclusion

This study of dynamic network backbones during general anesthesia provides novel information regarding the profile of network modules and their temporal deformation across various states of consciousness. These results suggest a complex network behavior comprised of constitutive and highly variable backbone structures that form a mosaic across distinct states of consciousness. Our findings also give further evidence for the disruption of frontal-parietal network elements during anesthetic-induced unconsciousness and the relative preservation of frontal-frontal networks. Further study is warranted on network backbones as a method of characterizing dynamic changes during normal and abnormal states of consciousness.
